# School functioning and internalizing problems in young schoolchildren

**DOI:** 10.1186/s40359-019-0365-1

**Published:** 2019-12-23

**Authors:** Marit Løtveit Pedersen, Solveig Holen, Stian Lydersen, Kristin Martinsen, Simon-Peter Neumer, Frode Adolfsen, Anne Mari Sund

**Affiliations:** 1 Regional Center for Child and Youth Mental Health and Child Welfare, Department of Mental Health, Faculty of Medicine and Health Sciences, Norwegian University of Science and Technology (NTNU), Trondheim, Norway; 2 Regional Centre for Child and Adolescent Mental Health, Eastern and Southern Norway (RBUP), Oslo, Norway; 3 Regional Centre for Child and Youth Mental Health and Child Welfare, UiT Arctic University of Norway, Tromsø, Norway; 4 Department of Child and Adolescent Psychiatry, St. Olavs University Hospital, Trondheim, Norway

**Keywords:** School functioning, Academic achievement, School adaptation, Anxiety, Depression, Children

## Abstract

**Background:**

Symptoms of anxiety and depression are common mental health problems in children and are often referred to as internalizing symptoms. Youth with such symptoms are at greater risk for poor academic achievement, school non-completion, and future mental health problems, all of which, lead to public health consequences and costs to society. The aim of the current study was to investigate associations between young school children’s internalizing symptoms and school functioning, as assessed separately by the teachers and the children.

**Methods:**

This study is a cross-sectional study including children (*N* = 750. 58% girls) from the ages of 8–12 years with elevated levels of self-reported symptoms of anxiety (MASC-C) and/or depression (SMFQ). Teachers reported the academic achievement, school adaptation (TRF) and internalizing symptoms (BPM-T) of the children**.** Associations were analyzed using linear regression analyses.

**Results:**

Both teacher-reported internalizing symptoms and children’s self-reported depressive symptoms were associated with poor academic achievement and school adaptation, while self-reported symptoms of anxiety were not. Symptoms of depression as assessed by the children were associated with teacher-rated internalizing symptoms, while self-reported symptoms of anxiety were not.

**Conclusion:**

We found negative associations between school functioning and internalizing symptoms, as assessed by both the teachers and the children. The dual findings strengthen the validity of these relationships. Thus, prevention of depressive and anxiety symptoms in children may lead to positive changes in school domains such as academic achievement and school adaptation. We also identified a negative association between teacher-rated internalizing symptoms and children’s self-report of depressive symptoms, indicating that teachers may have difficulties recognizing children with these symptoms.

**Trial registration:**

Clinical Trials NCT02340637, Registered on June 12, 2014, Retrospectively registered.

## Background

Good school functioning is important from a life course perspective, both for the individual, in terms of their health and school education [1] and for society, in terms of work employment and reduced societal costs [2]. Research also indicates that school functioning and mental health are associated and that problems in one domain may affect the other [1, 3, 4]. Internalizing symptoms (i.e. symptoms of anxiety and/or depression) are common psychological difficulties in children and adolescents [5], and several studies have reported that these children seldom receive professional care [6, 7]. Therefore, the association between school functioning and internalizing symptoms merits attention.

Academic achievement is important to every child and represents performance outcomes, i.e. the extent to which the child has accomplished educational goals [8]. The long-term implications of academic achievement can be both positive, e.g. academic career and employment possibilities when school performance is good, and negative, e.g. mental health problems, school dropout and unemployment when school performance is poor [1, 9, 10].

Successful adaptation to school may contribute to healthy cognitive and socio-behavioral development with good control of emotions and impulsive behaviors, and improved ability to cope with new situations and problems with peers or others [11, 12]. Several individual and contextual factors can affect and predict good school functioning. Individual factors such as intelligence [13], gender [14, 15] and beliefs about and values for the future [16] have been reported to affect school functioning. Supportive relationships with teachers and peers are also important contributors to good school functioning [17, 18]. In addition, socio-economic background, family support and parents’ educational level are strong predictors of successful learning and academic achievement [15, 17, 19, 20].

Results from systematic reviews of school performance from 2000 to 2014, mainly in primary and middle schools in Norway, Europe and the United States, indicate that girls adapt better to school and attain higher academic achievement than boys [14, 15, 21]. These gender differences also increase with age and seem to be relatively stable over time in several countries.

Studies indicate that 9–12% of all children have symptoms of anxiety and/or depression, commonly called internalizing problems, which clearly reduce their daily functioning, even when they do not qualify for a full diagnosis [22]. A Norwegian study among children aged 8–10 years (*N* = 9155) displayed prevalence rates in the population for psychiatric disorder ranging between 5.6–8.5% [6]. Anxiety and depressive disorders were the most common disorders. Previous research also indicates that some children have internalizing symptoms that go undetected [6].

Anxiety and depression often co-occur, and anxiety often precedes depression in youth [23, 24]. Anxiety and depression in childhood may also be precursors to other difficulties later in adolescence, such as low self-esteem and substance abuse [25], increased risk for poor academic achievement, school non-completion and future mental health problems [26–28], all of which may have serious public health consequences and costs for society [2].

The types of mental health problems reported seem to vary by gender. In general, girls report higher levels of internalizing symptoms and boys report higher levels of externalizing symptoms in primary school [5, 29]. In addition, these internalizing symptoms increase with age [5]. Hence, it is important to identify these children at an early stage; preventive efforts are imperative.

There is growing evidence of negative associations between internalizing symptoms and school functioning, suggesting that problems in one domain affect the other [1, 3, 4]. A cross-sectional study with children aged 7–14 years reported that children with anxiety disorders had lower levels of school functioning than children without an anxiety disorder [30]. Results from a longitudinal study indicated that children who were highly anxious in first grade scored significantly lower academically and higher on symptoms of anxiety and depression in eighth grade [31]. Other longitudinal studies showed that internalizing symptoms in sixth grade led to lower grade point averages (GPA) in the same school year and predicted more depressive and anxious symptoms in the following school year [32]. Lower levels of achievement and attainment at 20 years of age have also been reported [33]. Results from a meta-analysis highlighted that depressive symptoms, more than anxiety symptoms, led to poorer school functioning [34]. However, other studies have not replicated these associations. Duncan et al. [35] summarized analyses from six longitudinal studies and reported no significant associations between internalizing symptoms from the age of school entry and later academic achievement in elementary school. For some of the studies, these non-findings were also evident in middle school [35]. The authors emphasized that the conclusion might have been different with a clinical sample.

Other studies describe associations between early academic failure and internalizing symptoms later in life [1, 36]. Masten and colleagues [37] found that low academic competence in children aged 8–12 years predicted internalizing symptoms 10 years later. Another study, including children in the same age range, reported that low academic attainment in primary school predicted increased internalizing symptoms later in life [38]. There were no gender differences for either age range.

Regarding gender, a meta-analysis of studies of youths aged 8–18 years reported that associations between anxiety and school failure were stronger in girls [34]. In another population study, girls aged 12–14 years achieved better academic results but had poorer wellbeing and more depressive symptoms than the boys. Both genders, however, have been associated with the same levels of school-related stress [39].

Overall, associations between school functioning and levels of internalizing symptoms are documented bidirectionally in both cross-sectional and longitudinal studies [1, 33, 34]. In addition, studies indicate that success in school functioning may be a protective factor against later development of mental health problems [1]. Thus, targeting domains of internalizing symptoms or poor school functioning may have the potential to be preventive in other domains, such as later school dropout and unemployment.

Internalizing symptoms in children under 10–11 years of age are usually reported by their parents [33, 35]. Teachers’ reports are also often used when assessing internalizing symptoms in school [31, 40]. However, studies suggest that teachers may rate children’s difficulties significantly lower than do the children themselves [41, 42]. In fact, teacher ratings are, on average, lower than the ratings of other informants. Larsson and Drugli [43] also found that teacher-rated internalizing symptoms among Norwegian school children aged 6–13 years were significantly lower than the average reported in Rescorla et al.’s study [44] examining these symptoms in 21 countries. This may indicate lower real prevalence rates of internalizing symptoms among Norwegian school children; on the other hand, Norwegian teachers may underreport internalizing symptoms even more than do teachers in other countries. One explanation of the latter may be that overt and disruptive behaviors might manifest more in the classroom and may lead to teachers being less attentive to children with internalizing symptoms [45].

However, previous research suggests that different informants contribute unique information about a child’s problems [42, 46]. In other words, the informant discrepancies may reflect individual differences in how behavior is displayed based on context and the informants involved, and may reflect meaningful information on differences displayed by a child across different contexts [42, 46]. Thus, it is necessary to recognize the individual informants’ unique perspectives on internalizing symptoms. Because of this, it has been advocated that ratings from different informants are preferred [46, 47]. According to a recent review of assessments of psychosocial functioning in school-based services and research, informant discrepancies seem to be consistent across samples and studies over time [47]. This can make it difficult to draw conclusions as to the prevalence, outcomes and efficacy of interventions. There is a difference between measuring the characteristics of a subject and the different informants’ perceptions of those characteristics; in the latter case, preference should often be given to the reports of the different informants [48]. To further investigate the perceptions of different informants, there is a need to be more sensitive to the informant’s context [47].

To obtain a more complete and valid picture of how internalizing symptoms in young children are expressed in a school setting and how different informants view this issue, we examined both the teachers’ and young school children’s perceptions of the children’s internalizing symptoms. In addition, we investigated whether these symptoms affected the children’s academic achievement and school adaptation at this young age. The target group in the current study was school children aged 8–12 years with elevated symptoms of anxiety and/or depression, as assessed by the children themselves. The children were participating in an indicative intervention study, thus representing an at-risk population. This represents a targeted sample more seldom studied than general population or clinical samples.

Examining how symptoms of anxiety and depression as expressed at school are seen by both teachers and the children themselves may improve and broaden our understanding of at-risk children. By focusing especially on symptomatic sub-groups, we can determine if there are different relations between symptom level and school functioning, as seen by different informants. Previous research indicates that teachers are not always aware of children’s internalizing problems [42]. Thus, it is important to investigate possible discrepancies to see how these differences are manifested in a school setting. Based on this, we studied the associations between internalizing symptoms as assessed by the teachers and children with elevated symptom levels of anxiety and depression, as well as how their internalizing symptoms were associated with school functioning.

We first explored the characteristics of 8–12-year-old children with respect to school functioning in terms of academic achievement, school adaptation and internalizing symptoms, separately and for each gender. Internalizing symptoms in children were rated by the teachers as well as the children themselves, who answered questions about symptoms of anxiety and depression.

Then the following research questions were studied:

1. Are teacher-rated internalizing symptoms among young school children associated with academic achievement and school adaptation?
2. Are school children’s self-reported symptoms of anxiety and depression associated with academic achievement and school adaptation?
3. Are teacher-rated internalizing symptoms associated with school children’s self-reported symptoms of anxiety and depression?

We expected girls to perform better academically than boys, and to adapt better to school, as reported by their teachers. We also expected that more girls would report internalizing symptoms. Furthermore, we hypothesized that there were negative associations between internalizing symptoms and academic achievement, as well as school adaptation, as reported by the teachers. We then hypothesized that there would be negative associations between the children’s self-reporting of anxiety and depressive symptoms and their academic achievement and school adaptation. Finally, we expected that the association between teacher-rated internalizing symptoms and children’s self-reported symptoms of anxiety and depression would be relatively weak.

## Method

### Procedure

This study was part of a randomized controlled intervention study called Coping Kids: Early Intervention for Anxiety and Depression; The TIM study [49]. The aim of the TIM study was to investigate the effectiveness of a preventive group-based cognitive behavioral intervention called EMOTION, Coping Kids Managing Anxiety and Depression [50], which targets children aged 8–12 years with elevated levels of anxious and depressive symptoms. The intervention aims to reduce symptoms of anxiety and depression and possibly the likelihood of developing later disorders. Data used in the present study were based on the first data collection from the TIM-study. The Regional Committee for Medical and Health Research Ethics (2013/1909/REK South East) approved the study.

### Participants

A total of 36 primary schools from rural and urban areas in Norway participated in the study from 2014 to 2016. Approximately 7300 children from third to sixth grades (8–12 years of age) and their parents were informed about the study. Children were invited to participate if they (and/or their parents) considered themselves to have more sad or anxious feelings than their peers. After informed parental consent, 1692 children were invited to be screened using web-based questionnaires on symptoms of anxiety [51] and depression [52]. The screening took place in the schools, with teachers available to answer questions the children might have.

Of the screened children, 873 scored one standard deviation or more above a predetermined mean on self-reported anxiety and/or depression scales, and were invited to participate in the study. The cut-offs were based on Nordic and international studies in the relevant age group [53–55]. Of the 873 invited children, seven were excluded due to exclusion criteria: mental retardation, autism or severe behavioral disturbance. A total of 71 children were excluded randomly due to lack of resources (i.e. lack of group leaders implementing the intervention) and 45 dropped out before the intervention study started.

For included children, the children’s main teacher was asked to complete a web-based questionnaire about the children’s mental health and school functioning. In total, 750 children (58% girls) were rated by their teachers and thus, included in the present study. For more details on procedures, participants and sample size, see Patras et al. [49].

### Demographic information and age

Demographic information about the parents’ educational level was collected using data reported by the mothers.

Because of the relatively low level of social inequality in Norway, and the importance of the parents’ education level on both the children’s school functioning and their mental health [56, 57], we used the parents’ education level as a socio-economic status (SES) variable. We chose to use the mothers’ education level as a proxy for SES because more mothers than fathers had answered the questionnaires (*N* = 472 versus *N* = 91). Mothers’ education level (N = 472) indicated that approximately 9% had completed up to 2 years of high school, 22% had finished high school, 35% had attended up to 4 years of college or university and 33% had attended college or university for more than 4 years. Mothers’ education level was treated as a nominal variable in the analyses. Statistics Norway [58] reported that, of women between the ages of 25 and 49 years in the Norwegian population in 2017, approximately 17% had finished primary school, 2% had finished vocational school, 27% had finished high school, 38% had up to 4 years of college or university and 16% had attended college or university for more than 4 years. Thus, our sample had a skewed distribution of SES toward mothers with more education compared with the population data.

Regarding place of birth, 97% of the children, 93% of the mothers and 89% of the fathers were born in Norway (including up to 3% from Northern Europe) as reported by the mothers. We therefore did not include place of birth as a control variable in this study.

Month and year of birth were available for only 472 of the children. Therefore, class level was used as a proxy for age, which ranged from 8 to 12 years. Approximately 4% of the children were in third grade, 36% in fourth grade, 46% in fifth grade and 14% participated from the sixth grade. Generally, the children started in third grade the year they turned eight.

### Measures

#### Teacher’s report form (TRF)

Teacher-rated academic achievement and school adaptation were assessed using the Teacher’s Report Form (TRF)*,* a component of the Achenbach System of Empirically Based Assessment (ASEBA) [59]. We used the part of the TRF pertaining to academic achievement and adaptation to school. The teachers were asked to evaluate the children in four academic subjects—Norwegian, English, mathematics and social studies—and compare them with other children of the same age using a scale ranging from 1 to 5 (1 = far below average, 5 = far above average). A sum score was calculated based on the teachers’ answers on all four subjects, representing the academic achievement scale for the present study.

The TRF was also used to assess four characteristics that are considered important for school adaptation: [1] how hard he/she is working, [2] how appropriately he/she is behaving, [3] how much he/she is learning and [4] how happy he/she seems to be. The teachers were asked to compare the child’s characteristics with those of other children the same age on a scale ranging from 1 to 5 (1 = far below average; 5 = far above average), and a sum score was made representing the school adaptation scale.

The ASEBA system has shown good psychometric properties and has for decades been supported by research and feedback [44, 59]. In the present study, the internal consistency of the academic achievement scale was excellent (Cronbach’s alpha = 0.90) and the school adaptation scale was satisfactory (Cronbach’s alpha = 0.72).

### The brief problem monitor – teacher form (BPM-T)

Internalizing symptoms in children were assessed by the teachers using the Brief Problem Monitor – Teacher form (BPM-T) [60], a short 18-item version of the TRF which provides a uniform problem scale to assess both behavioral and internalizing symptoms of children in a school setting. In the present study, only the subscale for internalizing symptoms was used. The teachers rated the child during the previous 2 weeks on six items: (1) feeling worthless or inferior, (2) too fearful or anxious, (3) feeling too guilty, (4) self-conscious or easily embarrassed, (5) unhappy, sad or depressed and (6) worried. The items were rated on a scale ranging from 0 to 2 (0 = not true, 1 = sometimes true, 2 = very true). The sum score was used to represent internalizing symptoms as reported by teachers.

A systematic review of Scandinavian studies reported the reliability of the BPM-T total score to be satisfactory [61]. Internal consistency of the internalizing symptoms scale in the present study was good (Cronbach’s alpha = 0.82).

### The multidimensional anxiety scale for children (MASC-C)

Symptoms of anxiety were reported by the children on the Multidimensional Anxiety Scale for Children (MASC-C) [51]. This 39-item questionnaire assesses anxiety symptoms in children and adolescents between 8 and 19 years. The children rated each question on a scale from 0 to 3 (0 = never true about me, 1 = rarely true about me, 2 = sometimes true about me, 3 = often true about me) based on their experience in the past 2 weeks, and a sum score was calculated.

The MASC-C has shown high retest reliability [51, 62]. It has been evaluated in a Norwegian sample among 7–13-year-old treatment-seeking children and has favorable psychometric properties [63]. In the present study, internal consistency of the scale was good (Cronbach’s alpha = 0.84).

### The mood and feelings questionnaire-short version (SMFQ)

Symptoms of depression were reported by the children using the short version of the Mood and Feelings Questionnaire (SMFQ) [52]. This 13-item questionnaire, targeting children from 8 to 18 years, assesses cognitive, affective and behavioral-related symptoms of depression during the previous 2 weeks. The symptoms were rated from 0 to 2 (0 = not true, 1 = sometimes true, 2 = true). A sum score was calculated.

Previous studies indicated good psychometric properties on the Norwegian version of the SMFQ [64, 65]. In the current study, internal consistency of the scale was good (Cronbach’s alpha = 0.80).

### Statistical analyses

Descriptive statistics are reported as means and standard deviations (SD) for the variables in the total sample, as well as separately for each gender. Comparisons between the genders on the main independent variables were performed using Student’s t-test, and the Chi-square test was used for nominal variables.

Pearson correlations between the main variables —academic achievement, school adaptation, teacher-rated internalizing symptoms and children’s self-report on symptoms of anxiety and symptoms of depression— are also presented.

We used linear regression models with teacher-rated academic achievement and school adaptation, entered one at a time, as dependent variables. We carried out one set of analyses with teacher-rated internalizing symptoms as main independent variables, and one set of analyses with self-reported anxiety symptoms and self-reported depression symptoms as main independent variables. We also used linear regression models with teacher-rated internalizing symptoms as dependent variables, and children’s self-reported anxiety symptoms and self-reported depression symptoms as main independent variables. All analyses were adjusted for gender and class level.

Lastly, we replicated the analyses adjusting for mothers’ education level. The adjustment for mothers’ education level was done separately because it was reported for only 472 of the 750 participants. Two-sided *p*-values < 0.05 were considered statistically significant, and 95% confidence intervals (CI) are reported where relevant. Analyses were carried out using SPSS (v. 25; IBM SPSS, Armonk, NY, USA).

## Results

Descriptive data for dependent and independent variables, as well as gender differences, are presented in Table 1. More girls (58%) than boys participated in the study. On the main variables, girls reported higher levels of both academic achievement and school adaptation than boys, and scored higher on self-reported symptoms of anxiety and depression. Teachers, however, reported no gender differences on internalizing symptoms in the children.

**Table 1:** Mean and standard deviation (SD) for the main variables in the sample

	All	Girls	Boys	Gender differences
	N = 750	*N* = 435	*N* = 315	*p*-value
Academic achievement (T)^a^	11.81 (3.33)	12.11 (3.36)	11.38 (3.25)	0.003
School adaptation (T)^a^	12.15 (2.46)	12.86 (2.32)	11.17 (2.32)	< 0.001
Internalizing problems (T)^a^	2.57 (2.61)	2.46 (2.57)	2.73 (2.64)	0.166
Anxiety symptoms (S)^b^	63.60 (13.60)	66.53 (12.78)	59.55 (13.68)	< 0.001
Depression- symptoms (S)^b^	9.92 (4.89)	10.34 (4.92)	9.35 (4.79)	0.006

Note: Academic achievement = TRF/ASEBA (Range 4–20); School adaptation = TRF/ASEBA (Range 5–20); Internalizing problems = BPM-T (Range 0–12); Anxiety symptoms = MASC (Range 0–105); Depression symptoms = SMFQ (Range 0–26); (T) = Teacher rated; (S) = Self-report; ^a^ = High values indicate good performance; ^b^ = Higher values indicate more problems or symptoms

Pearson correlations showed significant associations between academic achievement and school adaptation as assessed by the teachers (see Table 2). Internalizing problems were negatively correlated with both academic achievement and school adaptation. Furthermore, there was a negative association between children’s self-report of depressive symptoms and school adaptation reported by the teachers.

**Table 2:** Pearson correlation matrix for the main variables in the sample *N* = 750

	Academic achievement (T)	School adaptation (T)	Internalizing problems (T)	Anxiety symptoms (S)	Depressive symptoms (S)
Academic achievement (T)	1				
School adaptation (T)	0.656*** < 0.001	1			
Internalizing problems (T)	−0.189*** < 0.001	− 0.286*** < 0.001	1		
Anxiety symptoms (S)	0.015 0.689	0.039 0.281	0.112*** < 0.001	1	
Depressive symptoms (S)	−0.068 0.061	−0.086** 0.018	0.160*** < 0.001	0.332*** < 0.001	1

Note: Academic achievement = TRF/ASEBA; School adaptation = TRF/ASEBA; Internalizing problems = BPM-T; Anxiety symptoms = MASC; Depression symptoms = SMFQ; (T) = Teacher rated; (S) = Self-report; *p < 0.05; **p < 0.01; ***p < 0.001

### Academic achievement

In the first regression model, teacher-rated academic achievement was the dependent variable and internalizing symptoms in children was the main independent variable (see Table 3). Results from the regression analyses indicated that, according to the teachers, internalizing symptoms were negatively associated with academic achievement (B = − 0.24, CI = − 0.33 to − 0.15, *p* < 0.001). Adjusting for mothers’ education level gave substantially the same results, even though mothers’ education level was positively associated with academic achievement.

**Table 3:** Regression model: Academic achievement as dependent variable and internalizing problems as main covariate

Independent variables	Academic achievement (T)	
	B (95% CI) N = 750	Adj. B (95% CI) N = 472
Internalizing problems (T)	−0.24*** (− 0.33 to − 0.15)	−0.22*** (− 0.33 to − 0.12)
Female gender	0.67* (0.19 to 1.14)	0.60* (0.02 to 1.18)
Class level	0.05 (−0.26 to 0.36)	0.069 (−0.31 to 0.45)
Mothers education level		0.89*** (0.60 to 1.17)

Note: B Regression coefficient, adjusted for gender and class level; Adj B: Adjusted for gender, class level and mother’s education level; *Academic achievement = TRF/ASEBA*; Internalizing problems = BPM-T; (T) = Teacher rated; *p < 0.05; **p < 0.01; ***p < 0.001

Results from the second regression model, with children’s self-reported symptoms of anxiety and depression as main independent variables and academic achievement as a dependent variable, are reported in Table 4. Self-reported symptoms of depression were negatively associated with academic achievement (B = − 0.058, CI = − 0.110 to − 0.006, *p* = 0.028). Self-reported symptoms of anxiety were not associated with academic achievement. Adjusting for mothers’ education level reduced the effect of depression to a non-significant level, and substantially reduced the effect of gender. This reduced effect of depression is not because of missing data on mothers’ educational level, but due to adding the mothers’ educational level to the model.

**Table 4:** Regression model: Academic achievement as dependent variable and symptoms of anxiety and depression as main covariates

Independent variables	Academic achievement (T)	
	*B (95% CI)* *N = 750*	*Adj. B (95% CI)* *N = 472*
Anxiety symptoms (S)	0.004 (−0.016 to 0.023)	0.006 (− 0.015 to 0.027)
Depression symptoms (S)	−0.058* (− 0.110 to − 0.006)	−0.042 (− 0.101 to 0.017)
Female gender	0.77** (0.27 to 1.26)	0.43 (−0.14 to 1)
Class level	0.04 (−0.27 to 0.36)	0.12 (−0.24 to 0.48)
Mothers education level		0.83*** (0.56 to 1.10)

Note: B Regression coefficient, adjusted for gender and class level; Adj B: Adjusted for gender, class level and the mothers education level; *Academic achievement = TRF/ASEBA;* Anxiety symptoms = MASC; Depression symptom = SMFQ; (T) = Teacher rated; (S) = Self-report; *p < 0.05; **p < 0.01; ***p < 0.001

### School adaptation

The results of regression analyses with internalizing symptoms in children as rated by teachers as the main independent variable, and school adaptation as the dependent variable, are reported in Table 5. Internalizing symptoms rated by the teachers were negatively associated with school adaptation (B = − 0.26, CI = − 0.32 to − 0.20, *p* < 0.001). Adjusting for mothers’ education level gave substantially the same results.

**Table 5:** Regression model: School adaptation as dependent variable and internalizing problems as main covariate

Independent variables	School adaptation (T)	
	B (95% CI) N = 750	Adj. B (95% CI) N = 472
Internalizing problems (T)	−0.26*** (− 0.32 to − 0.20)	−0.25*** (− 0.33 to − 0.17)
Female gender	1.62*** (1.30 to 1.94)	1.59*** (1.18 to 2.00)
Class level	0.10 (−0.11 to 0.32)	0.11 (−0.15 to 0.37)
Mothers education level		0.43*** (0.23–0.63)

Note: B Regression coefficient, adjusted for gender and class level; Adj B: Adjusted for gender, class level and mother’s education level; School adaptation = TRF/ASEBA; Internalizing problems = BPM-T; (T) = Teacher rated; *p < 0.05; **p < 0.01; ***p < 0.001

Results from regression analyses with children’s self-reported symptoms of anxiety and depression as the main independent variable and school adaptation as the dependent variable are reported in Table 6. Self-reported symptoms of depression were negatively associated with school adaptation (B = − 0.061, CI = − 0.097 to − 0.025, *p* < 0.001). Self-reported symptoms of anxiety were not associated with school adaptation. Adjusting for mothers’ education level produced substantially the same results.

**Table 6:** Regression model: School adaptation as dependent variable and symptoms of anxiety and depression as main covariates

Independent variables	School adaptation (T)	
	B (95% CI) *N* = 750	Adj. B (95% CI) *N* = 472
Anxiety symptoms (S)	−0.002 (− 0.015 to 0.011)	−0.006 (− 0.022 to 0.010)
Depression symptoms (S)	− 0.061*** (− 0.097 to − 0.025)	−0.058* (− 0.103 to − 0.012)
Female gender	1.76*** (1.41 to 2.11)	1.69** (1.25 to 2.13)
Class level	0.09 (−0.13 to 0.31)	0.18 (−0.10 to 0.47)
Mothers education level		0.43** (0.22 to 0.64)

Note: B Regression coefficient, adjusted for gender and class level; Adj B: Adjusted for gender, class level and the mothers education level; School adaptation = TRF/ASEBA; *Anxiety symptoms = MASC; Depression symptoms = SMFQ;* (T) = Teacher rated; (S) = Self-report; *p < 0.05; **p < 0.01; ***p < 0.001

### Internalizing symptoms as assessed by teachers and children

Results from regression analyses including teacher-rated internalizing symptoms as the dependent variable and children’s self-reported symptoms of anxiety and depression as the main independent variables are reported in Table 7. Self-reported symptoms of depression were associated with teacher-rated internalizing symptoms (B = 0.072, CI = 0.021 to 0.122, *p* < 0.01). Self-reported symptoms of anxiety were not associated with teacher-rated internalizing symptoms. Adjusting for mothers’ education level gave substantially the same results.

**Table 7:** Regression model: Internalizing problems as dependent variable and symptoms of anxiety and depression as main covariates

Independent variables	Internalizing problems (T)	
	*B (95% CI)* *N = 750*	*Adj. B (95% CI)* *N = 472*
Anxiety symptoms (S)	0.017 (0.001 to 0.036)	0.017 (0.002 to 0.036)
Depression symptoms (S)	0.072** (0.021 to 0.122)	0.071** (0.020 to 0.122)
Female gender	−0.455 (− 0.941 to 0.031)	−0.450 (− 0.937 to 0.038)
Class level	0.152 (− 0.158 to 0.462)	0.151 (− 0.159 to 0.462)
Mothers education level		−0.054 (− 0.289 to 0.181)

Note: B Regression coefficient, adjusted for gender and class level; Adj B: Adjusted for gender, class level and the mothers education level;*;* Internalizing problems = BPM-T; *Anxiety symptoms = MASC; Depression symptoms = SMFQ;* (T) = Teacher rated; (S) = Self-report;*p < 0.05; **p < 0.01; ***p < 0.001

## Discussion

The current study aimed to investigate associations between young children’s academic achievement and school adaptation and internalizing symptoms, as reported by children and their teachers. We also examined associations between teachers’ reporting of internalizing symptoms and the children’s self-report of symptoms of anxiety and depression.

The main findings indicated consistently that both teacher-reported internalizing symptoms and children’s self-report of depressive symptoms were negatively associated with academic achievement and school adaptation. Children’s self-reported anxiety symptoms were associated with neither teacher-rated academic achievement nor school adaptation. Furthermore, self-reported symptoms of depression were associated with teacher-rated internalizing symptoms, while self-reported symptoms of anxiety were not.

The descriptive data show that the total mean in academic achievement in our sample of children, who had elevated symptoms of anxiety and/or depression, was slightly lower than in the Larsson and Drugli [43] national population-based study of children from 6 to 13 years of age (Mean 2.9 versus 3.2). The total mean score in school adaptation was lower in our sample of at-risk children compared to the same national sample of children with a comparable age range (Mean 12.15 versus 17.19), as presented by Larsson and Drugli [43]. The children in our study might therefore be at risk for later problems regarding mental health and both achieving at school and school attendance [1, 36–38].

The girls in our study scored higher than the boys on teacher-rated academic achievement and school adaptation, which supports our hypothesis. These findings are in accordance with results from several previous studies [14, 21], which found that girls do better academically and adapt better to school than boys. Larsson and Drugli [43] found that girls aged 6–13 scored significantly higher than boys on teacher-reported total adaptive functioning, as well as on factors such as working hard, appropriate behavior and learning. They did not, however, find gender or age differences for academic performance on average. Our findings, using the same measurement (TRF) as Larsson and Drugli, however, indicate that girls have higher academic achievement and adapt better to school, as reported by their teachers. Among children with internalizing symptoms, boys might be more strongly affected than girls on domains such as academic performance and adaptation to school.

The children in our study were quite young and school demands are still low. In addition, the school system in Norway at the primary school level is generally not very competitive compared with other countries. When the children start middle school (13 years of age), grades, final exams and national tests will be introduced for the first time. The academic work may therefore be more challenging in middle school and high school. By that time, more academic problems may have emerged, which might also influence the level of internalizing symptoms.

In our study, the children were included based on a cut-off score that was one standard deviation or more above a chosen population-based mean on anxiety and/or depression scales. Thus, the sample might be relatively heterogonous with a broad range of symptoms. The relatively narrow standard deviations found in our sample, however, point to the opposite. Despite the young age of our sample, and the fact that this is not a clinical sample, the children in our study had higher levels of self-reported anxiety (Mean 63.60) than those in studies of children aged 7–13 years, both in a Norwegian clinical sample (Mean 57.00) [66]; and in a sample of referrals for anxiety with an anxiety diagnosis (Mean 55.22) [63];. Both these studies also used the MASC-C self-report instrument. Accordingly, the level of depressive symptoms in our sample, as measured by the SMFQ, was higher than in a large population-based study of 10–19-year-olds (Mean age = 13.8) from Middle Norway (Mean 9.92 vs. 4.50) [64];. This confirms that the children in our study represented an at-risk sample exhibiting elevated levels of subjective symptoms. Our findings might suggest that many of the children in our sample have high symptom levels which in many cases have not been detected. This underscores the need for early intervention for this group of children.

The girls in our study reported significantly higher levels of symptoms than the boys, both on self-reported symptoms of anxiety and depression, which also supports our hypothesis. These findings are in accordance with previous research [5, 29].

As hypothesized, the teachers reported fewer internalizing symptoms than the children themselves, as seen in Table 1. Even though the teachers knew that these children were recruited to the study based on elevated levels of internalizing symptoms, which could lead to judgement bias, the teachers scored many of the participating children relatively low on internalizing symptoms. The mean (Mean 2.57) in teacher-reported internalizing symptoms is in the lower quartile of the range of 0–12. The means on children’s self-reported symptoms of anxiety (Mean 63.60. Range 0–105) and of depression (Mean 9.92. Range 0–26) are close to the midpoints of the ranges. One reason for this might be that internalizing symptoms in general may be under-reported by teachers, as inner thoughts, feelings and mood are not easily observable [41, 42]. The teachers also reported no gender differences for internalizing symptoms. Similar findings were identified in a population-based study of children of the same age in Norway [43, 67]. It is possible that when it comes to internalizing symptoms as assessed by their teachers, the mental health of Norwegian schoolboys and schoolgirls is equally good. On the other hand, teachers may miss actual gender differences.

As hypothesized, internalizing symptoms as assessed by the teachers in our study were negatively associated with academic achievement and school adaptation for both genders, regardless of age and mothers’ educational level. Thus, the teachers did believe that those children with emotional symptoms also struggled at school. One possible source of bias is that the same informant reported on both measurements. However, the questions about school functioning and internalizing symptoms do not seem to overlap. Furthermore, the teachers knew the inclusion criteria for the children in the study.

Internalizing symptoms may also be expressed differently in younger children than in adolescents [68]. In children aged 8 to 12 years, depressive symptoms and anxiety are often expressed by an irritable mood and argumentative behavior. The teachers might interpret these symptoms as externalizing symptoms, rather than internalizing symptoms. In school, teachers are supposed to evaluate how the children are doing academically as part of their ordinary job and they are well trained in such evaluations. It is probably easier for teachers to evaluate how a child is doing when it comes to academic achievement and school adaptation than to know how a child feels internally. Internalizing symptoms may be difficult for teachers, health personnel or parents to identify [46], as the teacher may perceive an anxious or depressed child as calm and obedient and as a child who does not create any trouble or noise in a busy classroom. Nevertheless, as assessed by the teachers, there were strong associations between teacher-rated internalizing symptoms and how the children functioned at school.

Only children’s self-reported depressive symptoms, not anxiety symptoms, were associated with teacher-rated academic achievement and school adaptation. Furthermore, the associations were weak. We hypothesized that there would be an association between both symptoms of anxiety and depression as assessed by the children and how well these children performed academically and adapted to school. Nevertheless, the results indicate that the children with depressive symptoms did not do well at school. Depressive symptoms and thoughts, such as reduced ability to have fun, reduced ability to concentrate, restlessness, feeling they were not as good as other classmates, doing everything wrong and having little energy can cause these children to do less well at school than their capabilities suggest. These results are supported by Riglin’s [34] meta-analysis, which stated that depression was more consistently associated with poor school functioning than anxiety.

When mothers’ education level was added to the regression model, the association between children’s self-reported depression and academic achievement were reduced to a non-significant level. This indicates that having a mother with a high level of education might reduce the negative effects that depressive symptoms have on academic achievement. Earlier studies found that SES and especially educated parents were a predictor of increased learning [15, 17, 19, 20]. Those parents might, through learning strategies, structure and close supervision, mitigate the possible negative school implications of their children’s depressive symptoms.

As stated earlier, the children’s anxiety symptoms were not associated with academic achievement and school adaptation. Anxious children might work harder to meet the school’s requirements despite their worries which may or may not be related to school performance. Previous findings on this matter are contradictory [30, 31, 35]. Our study does not present a clinical sample, thus the children’s internalizing symptoms might not yet have a recognizable impact on school functioning and demands are quite low at this grade level. This might change when the children enter high school, where increased school demands combine with additional symptomatology. Furthermore, their anxiety symptoms might be related to issues other than school functioning.

Regarding inter-rater agreement, the children’s self-report of depressive symptoms, and not symptoms of anxiety, was associated with teacher-rated internalizing symptoms. This finding indicates that the teachers detect children with depressive symptoms more easily than those with anxiety symptoms. Depressed children can be perceived as less joyful, with diminished interest in activities, reduced motivation or energy and commitment to school work, tiredness, restlessness and irritable mood. The teacher might more easily observe these factors as such symptoms become more starkly contrasted with expected child behavior. On the other hand, it might be more difficult for teachers to differentiate between a pathological fear and a more natural fear of stressful school situations. Another possibility is that since these children seem to struggle academically, teachers can more easily identify them. When teachers try to support children who are struggling academically, they may find that some of these children have depressive symptoms. However, children with anxious symptoms who nevertheless do relatively well at school are not easily detected in the same way by their teacher. Caution should be exercised when teachers are used as informants to refer children to indicated interventions for anxiety.

As the results from the current study and previous research [46, 47] indicate outcomes do not always coincide when using different informants on internalizing symptoms. This doesn’t mean that either one is wrong; different informants offer different perspectives and observations across different contexts. If we assume that the teachers are best at evaluating the children’s school functioning, and that the children themselves know best how they feel, the model regarding academic achievement and school adaptation involving the two different informants is probably the most accurate one. Studies indicate that children’s self-report of anxious and depressive symptoms can be tailored to identify these symptoms [69, 70], as well as getting the subjective perspective from the children themselves. This indicates that young children who consider themselves anxious do not always struggle at school. However, we do not know whether these symptoms may influence the children’s academic achievement and school adaptation later in life.

### Strengths and limitations of the study

A strength of the study was the high response rate from both children and their teachers.

The present study was related to baseline data of an indicated preventive intervention trial that included an at-risk population of children with elevated symptom levels of anxiety and/or depression. This represents a sample more seldom studied than general population samples or clinical samples.

Although the children in our study had elevated symptom levels of anxiety and depression, and some of the children might have qualified for a diagnosis, the current sample was not a clinical population. The findings cannot therefore be generalized to a clinical sample.

The children and their parents were invited to the study based on the children’s self-evaluation of sad and anxious symptoms, which may have led to more children coming forward with their internalizing problems.

A strength of the study was the use of two informants—the teachers and the children— for the reporting of internalizing symptoms. Although they used different measures, multiple informants may indicate cross-methodological validity of the results: they have also demonstrated that results can differ by informants. However, adding parental information about the children’s internalizing symptoms and functioning to this study could have further strengthened the validity of the findings.

A limitation of this study was the cross-sectional design, which prevented us from making any causal inferences. Only longitudinal studies can reveal whether internalizing symptoms in young children are predictive of later disorders and later school functioning.

Another limitation may be that teachers who reported on the children in our study knew that the children had been included based on self-reported internalizing symptoms, which may have caused a possible bias in their judgement of severity. Despite this, the teachers reported fewer internalizing symptoms than did the children themselves.

Since we recruited children by using self-reported measures only, we might have missed out on children who could find it difficult to participate in studies like this (e.g. socially anxious and withdrawn children). To reach these children, and had the parents permitted it, we could have contacted school counsellors, psychologists or school nurses to nominate possible children, thus increasing representativity and possible making it easier for the teacher to detect child anxiety in this study. However, such an approach was not approved by the ethical committee in Norway. In addition, distinguishing between different types of anxiety problems could have provided a more differentiated understanding of how these difficulties are perceived by the teachers.

Nearly 70% of mothers had up to 4 years of post-secondary education, meaning that there was a skewed distribution of SES. This level of education is relatively high compared to the 2017 Statistics Norway population data [58]. The education levels of the mothers being a strong predictor of successful learning and academic achievement [19, 20], might have buffered the full negative effects of internalizing symptoms on school functioning in this sample.

However, there was a substantial amount of missing data regarding family background. One might assume that families with low SES or a non-Norwegian background were underrepresented among parents who participated. This might have influenced the results. In sum, our results are representative of a group of children displaying depressive and/or anxious symptoms and whose mothers are more highly educated than the rest of the population. Hence, the results do not necessarily apply to children with internalizing symptoms from a non-Norwegian background.

We did not measure intelligence level, or the children’s experience of family support or teacher support, information that is related to both emotional symptoms and school functioning [17, 19, 20]. Such information might have moderated the results.

## Conclusion

Both teacher-rated internalizing symptoms and children’s self-report of depressive symptoms were associated with academic achievement and school adaptation, independent of age and gender. Anxiety symptoms per se, as assessed by the children, were not associated with teacher-rated academic achievement or school adaptation. Children’s self-report of depressive symptoms were associated with teacher-rated internalizing symptoms, while children’s self-report of anxiety symptoms were not.

Teachers should be more aware of the symptoms of childhood depression—and especially anxiety—as these children often go undetected [6, 7]. More emphasis on such problems—how to observe, detect and alleviate them—could be implemented in teacher education programs. Schools in Norway are obligated to provide a healthy and safe environment for learning and development. This involves seeing each child’s needs, helping them and referring them to relevant agencies when needed. How a teacher perceives the children in the classroom might also influence how they facilitate their teaching of these children.

This study supports the importance of recognizing children’s subjective internalizing symptoms in the school context and addressing preventive efforts before they enter the challenging puberty years. Children with internalizing symptoms might be at risk for later psychiatric disorders and problems in different domains. Effective screening instruments in schools might be helpful for the detection of anxiety problems. Interventions in the school setting to improve internalizing symptoms, especially depressive symptoms, may have important long-term consequences for children and for society.

## Data Availability

The datasets generated and/or analyzed are not publicly available due to privacy policy but are available from the author on reasonable request.

## Abbreviations

ASEBA: Achenbach System of Empirically Based Assessment
BPM-T: Brief Problem Monitor – Teacher form
CI: Confidence interval
GPA: Grade point average
MASC-C: Multidimensional Anxiety Scale for Children
SD: Standard deviation
SES: Socio-economic status
SMFQ: Mood and Feelings Questionnaire-short version
TRF: Teacher’s Report form
